# Management of Primary Squamous Cell Carcinoma of the Pancreas: A Case Report

**DOI:** 10.3389/fsurg.2021.700229

**Published:** 2021-10-26

**Authors:** Danling Guo, Chao Chen, Sangying Lv, Guanzuan Wu, Wei Shi, Huaifeng Li, Hongjie Hu

**Affiliations:** ^1^Department of Radiology, Shaoxing People's Hospital, Shaoxing, China; ^2^Department of Radiology, Sir Run Run Shaw Hospital, Zhejiang University School of Medicine, Hangzhou, China; ^3^Department of Pathology, Sir Run Run Shaw Hospital, Zhejiang University School of Medicine, Hangzhou, China

**Keywords:** carcinoma, squamous cell, pancreas, response, treatment strategy, MRI

## Abstract

**Introduction:** Primary squamous cell carcinoma of the pancreas (SCCP) is a rare malignant tumor that has been reported in individual case reports only. The clinical data on primary SCCP treatment are limited. Therefore, the appropriate management strategy for this disease should be standardized.

**Case Presentation:** We present the case of a 63-year-old man admitted to our hospital for upper left abdominal pain for 2 months without weight loss or jaundice. Enhanced computed tomography (CT) and magnetic resonance imaging (MRI) of the abdomen revealed a mixed solid and cystic lesion in the pancreatic tail, measuring 35 × 37 mm in maximum diameter with enhancement. The patient was diagnosed with primary SCCP without metastasis, based on radiological and pathological findings. He did not receive neoadjuvant therapy postoperatively and was followed up by CT and MRI for 18 months without recurrence or metastasis.

**Result:** Complete resection is the most effective treatment for early stage primary SCCP. Abdominal MRI is an effective imaging tool for preoperative evaluation and postoperative follow-up of primary SCCP. The need for neoadjuvant therapy depends on various factors.

**Conclusion:** Primary SCCP is a tumor with poor prognosis. Risk factor control, early accurate radiologic evaluation, and individualized treatment strategies improve the quality of life and prolong the overall survival period of patients.

## Introduction

Primary squamous cell carcinoma of the pancreas (SCCP) is a rare malignant tumor since the pancreas typically lacks natural squamous cells. The pathophysiology is still unknown. According to a recent study, the incidence of SCCP is low, accounting for 0.5–5% of exocrine pancreatic tumors (1).

SCCP behaves more aggressively and the incidence rate of patients with SCCP has gradually increased over the years (1). It has a worse survival rate and a lower cure rate than other pancreatic cancers (2), which makes the treatment of this disease challenging. While there are several treatment strategies for this disease, such as surgery, chemotherapy, and/or radiotherapy, the clinical precise management plan for primary SCCP has not been documented systematically in literature (3–6). Some cases found that primary SCCP respond well to surgery, while others revealed that the disease respond to neoadjuvant chemoradiotherapy (4, 6).

The management strategy for this disease has only been documented in individual case reports. It is not clear which patients are eligible for complete surgical resection and whether neoadjuvant chemoradiotherapy is really needed. Here, we report a case of primary SCCP managed by complete resection without neoadjuvant therapy strategy. The patient was followed up for 18 months by CT and MRI without recurrence or metastasis and with favorable results. We sought to comment on the indications for complete resection and neoadjuvant therapy and analyze an appropriate therapy and evaluation strategy for primary SCCP.

## Case Description

A 63-year-old man was admitted to our hospital for upper left abdominal pain lasting 2 months, with no weight loss or jaundice. He was a non-smoker and had unremarkable medical and family medical histories. Physical examination findings were unremarkable.

Initial laboratory examinations, such as blood cell count, biochemical parameters, and liver and kidney function tests, were normal. Carcinoembryonic antigen and ferritin levels were slightly elevated. Other tumor markers were within normal range.

**Figure 1:**
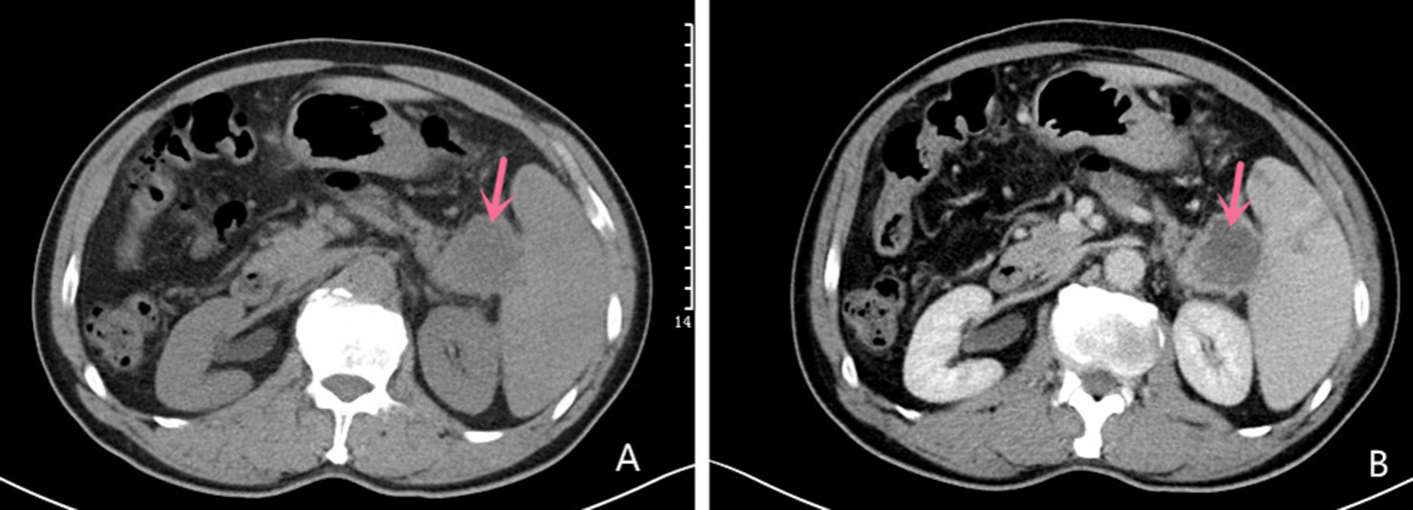
Computed tomography Imaging Computed tomography scan showing a mixed solid and cystic hypodense mass in the tail of the pancreas measuring 35 × 37 mm in the maximum diameter **(A)**; Contrast-enhanced CT imaging showing the mass with obvious enhancement **(B)**.

**Figure 2:**
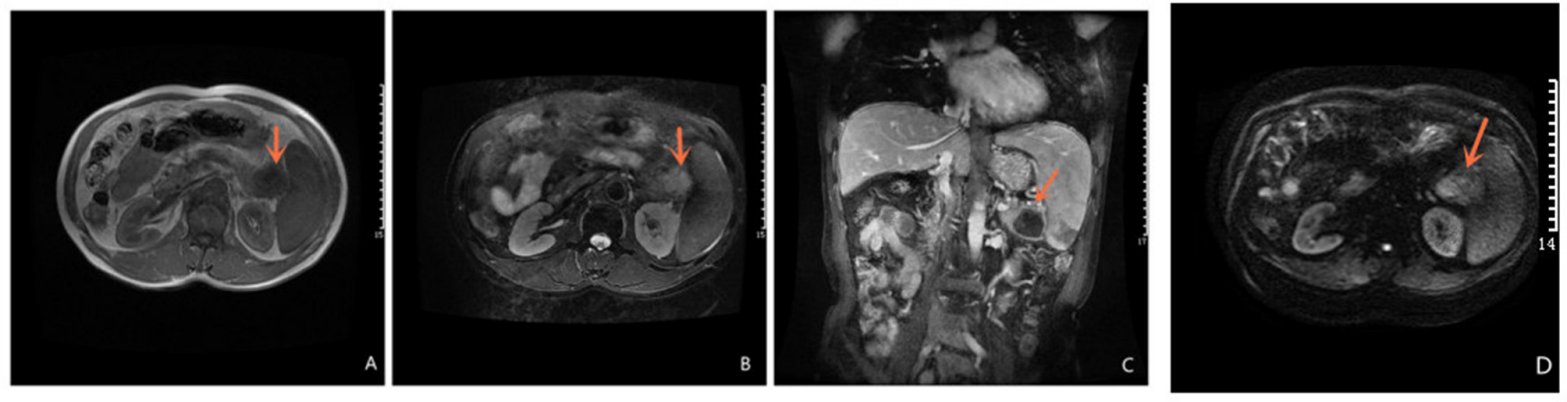
Magnetic Resonance Imaging Magnetic Resonance of abdominal revealed a mass in the tail of the pancreas. T1-weighted images is low signal **(A)** and T2 -weighted images is high signal **(B)**; Contrast-enhanced spin-echo T1-weighted MRI showed the lesion obvious enhancement **(C)**; The tumor showed restricted diffusion on Diffusion-Weighted imaging **(D)**.

Abdominal ultrasound demonstrated a hypoechoic mass, measuring 35 × 37 mm, in the pancreas' tail. Enhanced computed tomography (CT) of the chest, abdomen, and pelvis confirmed the presence of a low-density mass, measuring 35 × 37 mm in the maximum diameter, in the tail of the pancreas with enhancement. It adjoined the hilum of the spleen (Figure 1). The fat plane around the mass was blurred. The mass was not clearly demarcated from the splenic artery. Retroperitoneal lymph nodes were not observed. Magnetic Resonance imaging (MRI) of abdominal revealed a mass in the tail of the pancreas. The mass was low signal on T1-weighted images (Figure 2A) and high signal on T2-weighted images (Figure 2B). Contrast-enhanced spin-echo T1-weighted MRI showed the lesion obvious enhancement (Figure 2C). The tumor showed restricted diffusion on Diffusion-Weighted imaging (Figure 2D). There was a small artery passing through the tumor and merging into the splenic artery. The CT and MRI workup suggested a malignant tumor in the pancreatic tail. No mass was found in other parts of the patient's body after endoscopic and imaging examinations.

Due to non-specific laboratory and imaging findings, primary SCCP could not be differentiated with certainty from other malignant tumors of the pancreas. We advised him to undergo surgery considering the patient's stable condition (ASA class: Class 2) and the absence of vascular invasion. The patient agreed to be treated by complete resection after explaining to him the risks and possible complications in response to complete resection. Laparoscopic pancreatic body and tail combined splenectomy was performed under general anesthesia. Intraoperatively, there was a 37 × 33 mm mass in the tail of the pancreas. The mass was adhered to the hilum of the spleen and unclear boundary with the splenic artery. It is estimated that simple resection the mass is difficult, so we decided to separate the mass from the surrounding adhesion carefully and remove it with pancreatic body and tail and spleen. The surgery was performed smoothly without significant blood loss (100 ml) or further complications in the intraoperative course. On the second day after surgery, his C-reactive protein levels and neutrophil count were elevated. He was then administered intravenous anti-biotherapy (cefmetazole, 1 g BID). The Clavien Dindo Grade of our patient was Grade II and Comprehensive Complication Index was 20.9.

**Figure 3:**
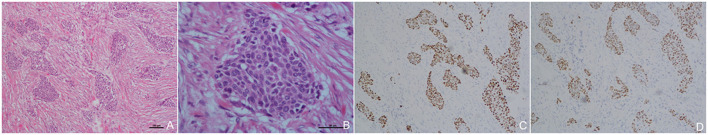
Histopathological and Immuno-histochemical images Low power image (HE × 40) of the carcinoma with squamous cell **(A)**; Higher power image with large squamous cell carcinoma nest and characterized by typical keratin pearls in histopathological assessments, without glandular component **(B)**. Immuno-histochemical showed p63 (+), p40 (+), and P63 and P40 as specific marker of squamous cell carcinoma **(C,D)**.

The entire tumor was sampled for histological examination, and the tumor margin was negative. On histopathological assessment, the mass was composed of large squamous cell carcinoma nests and characterized by the typical keratin pearls morphology (Figure 3A). The results revealed a well-differentiated SCCP with abundant cytoplasm and no glandular component (Figure 3B). Immuno-histochemical analysis showed CK-high (+), CK-low (+), CK5/6 (+), P63 (+), and P40 (+) (Figures 3C,D). The histological findings confirming the diagnosis of primary SCCP.

We advised him to undergo chemotherapy at our hospital after surgery; however, he declined the suggestion because the location was far from his home. His general condition was stable after surgery and then he was discharged home. The patient opted for periodic follow-up in the hospital near his home. After 18 months of follow-up, MRI and CT revealed no disease recurrence and metastasis.

## Discussion

Primary SCCP is a rare and fatal disease. Studies on pancreatic cancer epidemiology report that SCCP primarily affects males, blacks, and older patients (1, 4). Patients typically present with epigastric pain and weight loss (5). Other clinical presentations of primary SCCP depend on the location of the tumor (7). The pancreas normally lacks natural squamous cells (8). Therefore, an extensive workup to exclude metastases is required before diagnosing primary SCCP (1, 4). In our patient, imaging modalities, such as head, neck, chest, and total abdominal CT scans, as well as an endoscopic examination of the gastrointestinal tract, showed no other candidate primary site. We acquired specimens at multiple levels, and repeated analysis revealed that the tumor was characterized by a large squamous cell carcinoma nest and squamous cells with keratin pearls. On immune-histochemical staining, the tumor cells were positive for CK5/6, which supported the diagnosis. The tumor most probably originated from aberrant squamous cells.

There is no standard management strategy for this disease owing to the limited current clinical data on SCCP treatment (3). Therefore, the appropriate management strategy for this disease should be standardized. According to previous research, complete surgical resection is considered the only effective and curative treatment (9, 10). Patients who underwent curative resection (21 months) had superior median overall survival (OS) compared to patients who only received adjuvant therapy (5 months) (10). Therefore, our patient underwent a laparoscopic pancreatic body and tail combined splenectomy under general anesthesia and was followed up for 18 months without recurrence or metastasis. Resection improved the survival rate of patients with stages I, IIA, and IIB tumors (9, 11). So it is necessary to accurately determine the stage of a pancreatic tumor before resection (5). Our patient had a tumor localized in the pancreatic tail, with a maximum diameter of <4 cm and without lymph node metastases or distant metastases on CT and MRI. This confirmed that it was an early-stage tumor (stage IIB). Resectable tumors are those without peripheral vascular involvement or contour deformity based on the criteria of the National Comprehensive Cancer Network (6). Although the boundary between the mass and splenic artery was unclear, its contours were not irregular. Therefore, the tumor was suitable for surgical resection. The patient benefited significantly from complete resection. Patient in our study had early-stage primary SCCP with no comorbidities, which made it possible to properly evaluate the efficacy of complete resection with minimal confounding factors.

While surgery improves median OS and has a good effect, only 15–25% of patients are suitable for surgical resection at the time of initial diagnosis because of the high percentage of patients with advanced disease (12). This shows the importance of detecting this cancer in early stages. Abdominal CT is the preferred imaging modality to assess the extent of tumor invasion and determine the feasibility of surgery (13). However, CT has its drawbacks, especially in the differentiation of small masses from the background pancreas (6). MRI can be used to aid the diagnosis and identify metastasis. To our knowledge, this is the first primary SCCP case in which both preoperative evaluation and postoperative follow-up were performed using MRI. Our case demonstrated new vessel formation and a higher attenuation of the tumor on contrast-enhanced MRI, although this has not been reproduced. The sensitivity, specificity and accuracy of MRI for diagnosis of pancreatic mass were higher than CT (93, 89, and 90 vs. 90, 87, and 89%) (14). MRI is particularly useful in evaluating small or isoattenuating masses. Therefore, it is reasonable to perform MRI before treatment option in those patients who potentially underwent curative resection on initial staging CT. Furthermore, MRI is useful in evaluating liver metastasis at an early stage. Therefore, it is also important for postoperative follow-up.

Neoadjuvant chemo-radiotherapy is another commonly used preoperative or postoperative treatment (15). Neoadjuvant chemotherapy or radiotherapy is essential in treating other squamous cell cancers, such as rectal, anal, and esophageal squamous cell carcinoma (5). However, few studies have fully commented on the efficacy of neoadjuvant therapy in primary SCCP. Some studies suggested that neoadjuvant chemo-radiotherapy is important and required for primary SCCP (4). They also argue in favor of neoadjuvant chemotherapy (or chemo-radiotherapy) to patients with newly diagnosed, borderline resectable or unresectable SCCP to promote down staging and increase the chance of a successful future resection (6, 16). Patients with locally advanced tumors who received neoadjuvant chemo-radiotherapy had better OS than those who did not (4). However, some studies suggest that neoadjuvant therapy may be unnecessary if the primary SCCP is resectable (3). Tella et al. demonstrated that postoperative radiotherapy and/or chemotherapy did not improve the median OS in patients with stage I-II disease. In addition, palliative treatment failed to decrease mortality for patients with advanced disease (10). Our patient did not receive neoadjuvant therapy after surgery because he was in the early stage of the disease. His general condition was stable with no recurrence or metastasis at the 18-month follow-up, which further indicating that the effect of neoadjuvant chemo-radiotherapy is uncertain and need to be further studied. The need for neoadjuvant therapy after surgery depends on various factors, including the grade and margin of the tumor and vascular involvement. Only 20% of patients receive chemotherapy after curative surgery (5).

Despite the individualized treatment strategies, primary SCCP has maintained a high mortality rate and poor prognosis. Risk factor control is also necessary. The main risk factors for primary SCCP include chronic inflammation states and incorrect pancreatic duct stent placement (1, 17). Focusing on screening these high-risk patients might help in the early detection of primary SCCP. However, our patient had no history of chronic pancreatitis, and the stent was inserted preoperatively.

## Conclusion

In summary, primary SCCP is a rare and aggressive pancreatic malignancy. Based on our findings, the effective management of patients with SCCP need be further studied.

## Data Availability Statement

The original contributions presented in the study are included in the article/Supplementary Material, further inquiries can be directed to the corresponding authors.

## Ethics Statement

Written informed consent was obtained from the individual(s) for the publication of any potentially identifiable images or data included in this article.

## Author Contributions

DG performed the majority of the writing. CC and SL reviewed the literature and contributed to manuscript drafting. HL and GW prepared the radiology image. HH was responsible for the revision of the manuscript for important intellectual content. WS analyzed and interpreted the pathology findings. All authors issued final approval for the version to be submitted.

## Funding

This study was approved by Key Laboratory of Functional Molecular Imaging of Tumour and Interventional Diagnosis and Treatment of Shaoxing City.

## Conflict of Interest

The authors declare that the research was conducted in the absence of any commercial or financial relationships that could be construed as a potential conflict of interest.

## Publisher's Note

All claims expressed in this article are solely those of the authors and do not necessarily represent those of their affiliated organizations, or those of the publisher, the editors and the reviewers. Any product that may be evaluated in this article, or claim that may be made by its manufacturer, is not guaranteed or endorsed by the publisher.
