# Supplementary material for: Management of Primary Squamous Cell Carcinoma of the Pancreas: A Case Report
Source: Front Surg. 2021 Oct 26;8:700229. doi: 10.3389/fsurg.2021.700229 (PMC8575694; doi:10.3389/fsurg.2021.700229)
Supplement: Supplementary file 1 [file Table_1.PDF]

Timeline	
Time	Diagnosis and Treatment
2019/8/1	Admitted to our hospital
2019/8/2	Enhanced magnetic resonance imaging of the abdomen；Chest computed tomography(CT)
2019/8/3	Enhanced CT of the abdomen and pelvis
2019/8/6	Head and neck CT
2019/8/7	Laparoscopic pancreatic body and tail splenectomy under general anesthesia
2019/8/13	Postoperatively enhanced CT of the upper abdomen
2019/8/13	Pathological results: Primary squamous cell carcinoma of the pancreas
2019/8/16	Discharged from hospital
2019/9/5	One month following surgery, enhanced CT of the upper abdomen
2019/10-2021/5	Periodic follow up at the hospital near his home
